# Ratiometric fluorescent sensors based on MXene quantum dots: linking photophysics, architecture, and multi-analyte food detection

**DOI:** 10.1039/d6ra02430k

**Published:** 2026-05-20

**Authors:** Mohamed Abu Shuheil, Fatima Mayn Fadl, Israa Abdulhameed Ahmad, Maharshikumar B. Shukla, Rekha M. M., Priyanka Sharma, Bakirov Juma, Murodjon Yaxshimuratov, Shayan Mahmoodi

**Affiliations:** a Faculty of Allied Medical Sciences, Hourani Center for Applied Scientific Research, Al-Ahliyya Amman University Amman Jordan; b Department of Pharmacy, College of Pharmacy, The Islamic University Najaf Iraq; c Department of Anesthesia Techniques, Health and Medical Techniques College, Alnoor University Mosul Iraq; d Department of Chemistry, Faculty of Science, Gokul Global University Sidhpur Gujarat India; e Department of Chemistry and Biochemistry, School of Sciences, JAIN (Deemed to be University) Bangalore Karnataka India; f Department of Forensic Science, University Institute of Allied Health Science, Chandigarh University Mohali Punjab India; g Department of Preschool and Primary Education, Termiz University of Economics and Service Termez Uzbekistan; h Urgench State University Urgench Uzbekistan Uzbekistan; i Young Researchers and Elite Club, Tehran Branch, Islamic Azad University Tehran Iran sh.mahmoodiacademic@gmail.com

## Abstract

MXene quantum dots (MQDs) have recently emerged as highly promising nanomaterials for advanced food safety sensing due to their unique electronic structure, rich surface chemistry, and tunable photoluminescence. In this review—a comprehensive overview of MQD-based fluorescent and ratiometric platforms for food analysis—we systematically bridge fundamental photophysical mechanisms with architecture-level sensor design and real-world analytical performance. The discussion begins with the photophysical origins of MQD fluorescence, emphasizing the roles of quantum confinement, surface terminations, defect states, and transition metal d-orbitals in shaping emissive behavior. Building on this foundation, we analyze design strategies for single-emission and ratiometric sensing architectures, highlighting intrinsic and hybrid approaches that enhance signal reliability, self-calibration, and resistance to matrix interference. Recent advances in detecting biogenic amines, antibiotics, nitrite, and multi-analyte targets in complex food systems are critically evaluated, with particular attention to smartphone-integrated and dual-modal platforms. Finally, key translational challenges—including scalability, reproducibility, and regulatory integration—are outlined. Overall, MQD-based ratiometric sensors represent a transformative direction for rapid, robust, and field-deployable food safety monitoring technologies.

## Abstract 2

MXene quantum dots enable dual-emission ratiometric sensing, where analyte interactions alter signal ratios. This allows sensitive, selective, portable detection of multiple contaminants for rapid on-site food safety monitoring.

## Graphical abstract

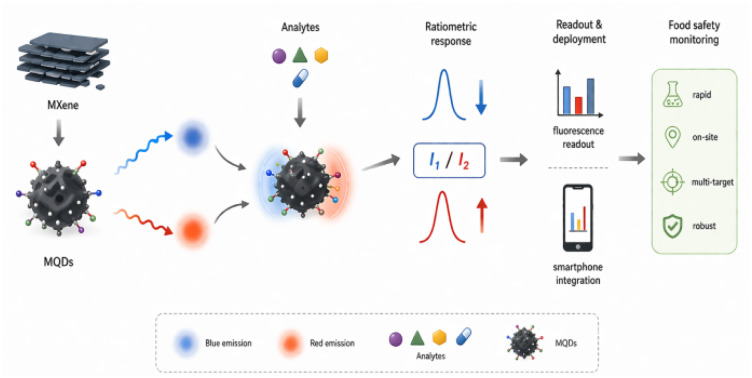

## 1. Introduction

Ensuring the safety and quality of food products remains a global priority as food supply chains become increasingly complex and geographically extended.^1–3^ Contamination by biogenic amines, antibiotic residues, nitrite, heavy metals, and other hazardous species poses significant risks to public health and regulatory compliance.^4–6^ Conventional analytical techniques, including chromatography and mass spectrometry, provide high accuracy but often require sophisticated instrumentation, extensive sample preparation, and trained personnel. These limitations have stimulated intense interest in rapid, sensitive, and portable sensing technologies capable of on-site food monitoring.^7–9^ Among emerging optical approaches, fluorescent nanomaterials have attracted particular attention due to their high sensitivity, fast response, and potential for miniaturization.^10–13^

In recent years, MXene quantum dots (MQDs) have emerged as a distinctive class of fluorescent nanomaterials with considerable promise for analytical sensing. Derived from two-dimensional MXene parent phases, MQDs combine ultrasmall size with metallic or semi-metallic electronic characteristics and abundant surface terminations.^14,15^ Unlike conventional semiconductor quantum dots or carbon dots, MQDs exhibit a hybrid photophysical nature arising from the interplay of quantum confinement, surface-state emission, defect chemistry, and transition metal d-orbital contributions. This unique electronic structure enables tunable photoluminescence, large Stokes shifts, and strong environmental responsiveness, all of which are highly desirable for chemical and biological sensing.^16,17^

The growing interest in MQDs for food safety analysis is driven by several intrinsic advantages. First, their rich surface chemistry—including –O, –OH, –F, and other functional groups—facilitates versatile functionalization and selective analyte recognition. Second, their high surface-to-volume ratio enhances interaction with target molecules, improving sensitivity. Third, MQDs can be engineered to exhibit excitation-dependent and multi-center emission behavior, which naturally supports ratiometric sensing strategies.^18–20^ These features position MQDs as more than simple fluorescent probes; rather, they function as programmable optical transducers that can be integrated into sophisticated sensing architectures.

Ratiometric fluorescence sensing has gained prominence as a robust strategy to overcome the limitations of single-emission probes. By encoding analytical information in the ratio between two optical signals, ratiometric platforms inherently compensate for fluctuations in excitation intensity, probe concentration, optical path length, and environmental variability. This self-referencing capability is particularly valuable in complex food matrices, where turbidity, pH variation, and background interference frequently compromise intensity-based measurements.^21–24^ MQDs are especially well suited for ratiometric design because their heterogeneous emissive landscape—originating from surface states, defects, and quantum-confined domains—can be deliberately harnessed to generate dual or multi-channel emission.

Despite rapid progress, the MQD sensing field remains conceptually fragmented. Many studies focus either on material synthesis or on isolated sensing demonstrations, often without establishing a clear connection between fundamental photophysics, architectural design principles, and real-world analytical performance.^25,26^ Moreover, while numerous reports describe MQD-based probes for specific food contaminants, a unified framework that links emission mechanisms to ratiometric platform engineering and translational considerations is still lacking. Addressing this gap is essential for guiding rational design and accelerating the transition from proof-of-concept systems to deployable technologies.^27–30^

This review aims to provide the comprehensive and integrative overview of MQDs in fluorescent and ratiometric food safety sensing. Rather than treating MQDs solely as novel materials, we adopt a systems-level perspective that connects photophysical origin, architecture-level engineering, and performance-driven applications. We begin by examining the fundamental mechanisms governing MQD fluorescence, including quantum confinement effects, surface termination chemistry, defect-mediated emission, and the role of transition metal electronic structure. Building on this foundation, we analyze the conceptual logic of single-emission *versus* ratiometric architectures, highlighting strategies for dual-channel engineering, intrinsic ratiometric behavior, and hybrid nanostructure design.

Subsequently, recent advances in MQD-based detection of key food contaminants—including biogenic amines, antibiotics, nitrite, and multi-analyte targets—are critically evaluated, with emphasis on analytical figures of merit, operational robustness, and smartphone-assisted platforms. Finally, we discuss the major translational challenges that must be addressed for practical deployment, including scalability, reproducibility, standardization, and regulatory acceptance.

By bridging fundamental photophysics with smart sensing architecture and real-world performance, this review seeks to clarify design principles, identify current limitations, and outline future opportunities for MQD-enabled food safety monitoring. The continued convergence of MXene chemistry, fluorescence engineering, and portable analytical technologies is expected to drive the development of next-generation sensing systems capable of rapid, reliable, and field-deployable food quality assessment.

## 2. Photophysical origin of fluorescence in MQDs

### 2.1. Quantum confinement and size-dependent electronic structure in MQDs

MQDs exhibit photoluminescence behavior that is fundamentally governed by quantum confinement effects arising from their ultrasmall lateral dimensions and reduced thickness. When the characteristic size of MXene fragments approaches or falls below the exciton Bohr radius, the continuous electronic bands of the parent MXene collapse into discrete energy levels. This discretization alters the density of states near the Fermi level and enables radiative recombination pathways that are absent in bulk MXenes, which are typically metallic or semi-metallic in nature.^31,32^

In MQDs, quantum confinement induces a widening of the effective bandgap as particle size decreases, leading to size-dependent emission energies. Experimental and theoretical studies indicate that lateral dimension plays a more dominant role than thickness in modulating the optical bandgap, owing to the strong in-plane electronic delocalization inherited from the MXene lattice. As a result, MQDs often display excitation-dependent emission behavior, reflecting a distribution of quantum-confined domains with varying sizes and edge configurations rather than a single uniform emissive center.

Importantly, quantum confinement in MQDs does not simply replicate the behavior observed in conventional semiconductor quantum dots.^33–35^ The presence of metallic d-electrons, anisotropic conductivity, and surface terminations introduces additional complexity into the electronic structure. The confined states in MQDs often retain partial metallic character, resulting in hybridized electronic states that lie between classical semiconductor excitons and surface-localized emissive centers. This hybrid nature is a key distinguishing feature that influences radiative lifetimes, Stokes shifts, and emission stability.

Furthermore, confinement-induced symmetry breaking at edges and corners generates localized states that act as radiative recombination centers. These edge-dominated states are particularly significant in MQDs due to their high edge-to-basal-plane ratio.^36,37^ Consequently, the photoluminescence of MQDs is best described as an emergent property arising from the interplay between quantum confinement, edge-state formation, and residual metallic conductivity, rather than a simple band-to-band transition model.

**Fig. 1:**
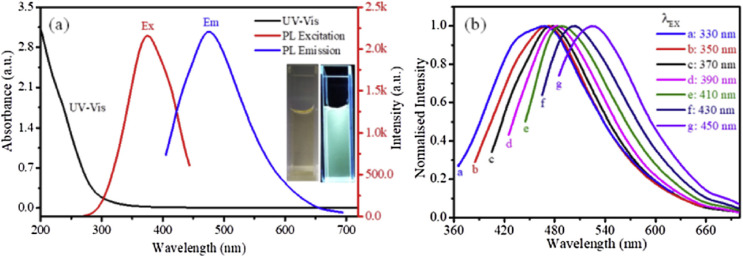
(a) UV-vis absorption, PL excitation, and emission spectra of Nb_2_C QDs in aqueous solution. (b) Normalized excitation-dependent emission spectra (330–450 nm), highlighting size- and surface-state-mediated photoluminescence behavior. Adapted with permission from ref. 37. © 2020 Elsevier B.V.

The optical characteristics of Nb_2_C QDs shown in Fig. 1 illustrate general photophysical features widely observed across MQD systems. The monotonic increase in UV absorption toward shorter wavelengths and the absence of distinct visible absorption peaks (Fig. 1a) are consistent with the transition from quasi-continuous electronic bands in bulk MXenes to discrete electronic states under strong lateral quantum confinement. The excitation-dependent emission behavior (Fig. 1b) further reflects the presence of multiple emissive domains arising from size dispersion and heterogeneous edge configurations. Similar excitation-dependent fluorescence has been reported for several MQD compositions, suggesting that heterogeneous surface and edge states represent a common emission mechanism in this material class. Collectively, these observations highlight a general design implication: controlling MQD lateral size distribution and edge chemistry is critical for achieving predictable emission wavelength and fluorescence stability in MQD-based sensing platforms.

### 2.2. Role of surface terminations in modulating radiative recombination pathways

Surface terminations are intrinsic to MXenes and remain a defining feature after their transformation into quantum dots. Functional groups such as –O, –OH, –F, and –Cl strongly influence the electronic structure of MQDs by altering surface potential, charge distribution, and local symmetry. These terminations introduce surface states within the bandgap or near-band-edge regions, which can act either as radiative emissive centers or non-radiative trap states depending on their chemical nature and spatial distribution. Oxygen-containing terminations generally promote radiative recombination by stabilizing surface-localized excitons and enhancing orbital overlap between metal d-states and ligand p-states. In contrast, fluorine-rich surfaces tend to increase non-radiative relaxation pathways due to stronger electronegativity and deeper trap formation. The balance between these competing pathways determines emission intensity, quantum yield, and photostability.^38,39^

Unlike passivated semiconductor quantum dots, where surface ligands are externally introduced, MQD terminations are structurally integrated into the lattice. This integration results in strong electronic coupling between the surface and the core, making surface chemistry a primary determinant of photophysical behavior rather than a secondary modification. Subtle changes in termination density or composition can therefore induce significant shifts in emission wavelength and lifetime.

Additionally, heterogeneous termination distributions lead to multiple emissive centers coexisting within a single MQD population. This heterogeneity contributes to excitation-wavelength-dependent fluorescence and broad emission bands commonly observed in MQDs. Importantly, these surface-induced emissive states are highly sensitive to local electronic perturbations, positioning MQDs as inherently responsive fluorescent materials even before deliberate sensor design is introduced.^39–41^ Thus, surface terminations in MQDs should be viewed not merely as chemical decorations but as active electronic components that define the radiative landscape of the system. Understanding and controlling termination chemistry is therefore essential for interpreting and predicting MQD photoluminescence at a fundamental level.

**Fig. 2:**
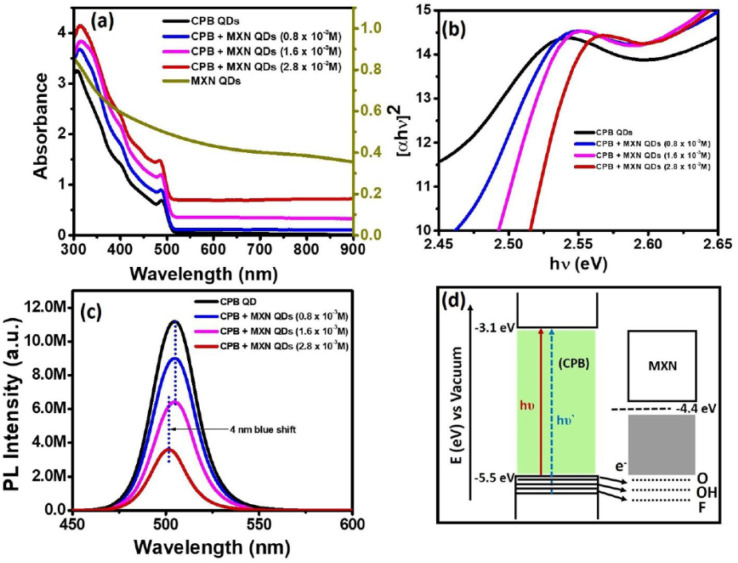
(a) Absorbance spectra of CsPbBr_3_ QDs, MXN QDs, and CPB–MXN composites showing MXene-induced spectral modification. (b) Corresponding Tauc plots highlighting excitonic blue shift. (c) Steady-state PL spectra (*λ*_ex_ = 410 nm) demonstrating concentration-dependent quenching. (d) Schematic band alignment illustrating interfacial charge transfer and termination-mediated electronic coupling in the QD/QD heterostructure. Adapted with permission from ref. 40. © 2020 American Chemical Society.

The CPB–MXN QD/QD heterostructure shown in Fig. 2 provides a representative example of how MXene surface terminations influence interfacial electronic interactions in hybrid systems. While the fundamental absorption profile of CsPbBr_3_ QDs remains largely preserved, the gradual blue shift of the excitonic edge with increasing MXN content (Fig. 2a and b) indicates that MQDs can perturb the electronic environment of adjacent semiconductor nanocrystals through termination-induced surface potentials. The concentration-dependent quenching observed in the steady-state PL spectra (Fig. 2c) further demonstrates efficient interfacial charge transfer enabled by electronically active MXene surfaces. Time-resolved measurements (Fig. 2d) confirm accelerated carrier relaxation in the composite system, supporting the role of MQDs as charge acceptors. More broadly, this behavior illustrates a key design principle for MQD-based hybrid sensors: engineered surface terminations can regulate interfacial charge transfer and exciton dynamics, thereby enabling controllable fluorescence modulation for sensing applications.

### 2.3. Defect states, edge chemistry, and their contribution to emissive centers

Defects and edge structures play a central role in the fluorescence of MQDs due to their high surface-to-volume ratio and truncated crystal lattice. Structural defects such as vacancies, lattice distortions, and incomplete metal–carbon bonding introduce localized electronic states that can serve as emissive centers. These defect-derived states often dominate photoluminescence, particularly when band-edge recombination is weak or suppressed.

Edges in MQDs represent regions of broken symmetry and altered coordination environments. Metal atoms at edges exhibit unsaturated bonds and modified oxidation states, leading to localized electronic levels that differ markedly from those in the basal plane. These edge states can trap charge carriers and facilitate radiative recombination with relatively long lifetimes, contributing to stable fluorescence emission. Edge chemistry further modulates these effects through interactions with terminating groups or adsorbed species. For instance, oxygenated edges tend to stabilize emissive defect states, whereas fluorinated edges may enhance non-radiative decay.^42,43^ The diversity of edge environments within a single MQD population gives rise to multiple emissive pathways, often manifested as broad and asymmetric emission spectra.

Notably, defect-related emission in MQDs differs from that in carbon dots, where fluorescence is largely attributed to molecular fluorophores or surface states alone. In MQDs, defect states are embedded within a conductive or semi-conductive framework, enabling electronic communication between defect sites and the underlying lattice. This coupling influences charge migration, recombination kinetics, and emission intermittency. From a photophysical perspective, defects and edges should not be regarded as undesirable imperfections but as intrinsic and functionally significant features.^43,44^ Their controlled generation and stabilization offer a route to tailoring MQD fluorescence without altering overall composition or size, highlighting their importance in the fundamental understanding of MQD photoluminescence.

### 2.4. Influence of metal d-orbitals and MXene core composition on optical transitions

A defining distinction between MQDs and conventional fluorescent nanomaterials lies in the active involvement of transition metal d-orbitals in optical transitions. The metallic core of MQDs contributes d-electron states near the Fermi level, which hybridize with carbon and surface ligand orbitals to form complex electronic structures. These hybridized states participate directly in absorption and emission processes. The choice of transition metal (*e.g.*, Ti, Nb, V, Mo) significantly influences the energy distribution of d-states and, consequently, the photophysical properties of MQDs. Metals with partially filled d-orbitals introduce mid-gap states that facilitate low-energy optical transitions, whereas metals with more delocalized d-electrons may suppress radiative recombination due to enhanced non-radiative relaxation.^45,46^

This metal-dependent behavior results in distinct absorption profiles, emission wavelengths, and excited-state dynamics across different MQD compositions. Importantly, optical transitions in MQDs often involve charge transfer between metal d-states and ligand or surface states rather than pure excitonic recombination. Such metal-to-ligand or ligand-to-metal charge transfer transitions contribute to large Stokes shifts and excitation-dependent emission.

The presence of d-orbitals also introduces strong spin–orbit coupling effects, which can influence intersystem crossing and triplet-state formation. These effects may play a role in delayed fluorescence or phosphorescence-like behavior reported in certain MQD systems. Overall, the involvement of metal d-orbitals endows MQDs with photophysical characteristics that bridge metallic and semiconducting regimes.^47,48^ This hybrid electronic nature underpins their unique fluorescence behavior and differentiates them fundamentally from carbon-based or purely semiconductor quantum dots.

### 2.5. Excited-state dynamics, charge carrier relaxation, and fluorescence stability

The fluorescence performance of MQDs is ultimately governed by excited-state dynamics, including charge carrier generation, migration, trapping, and recombination. Upon photoexcitation, electrons and holes are generated within hybridized electronic states and undergo rapid relaxation through both radiative and non-radiative pathways. Carrier relaxation in MQDs is strongly influenced by the coexistence of conductive domains and localized emissive states. Excited carriers can migrate through the MXene lattice before being trapped at surface states, defects, or edges where radiative recombination occurs. This migration–trapping mechanism explains the relatively long fluorescence lifetimes observed in some MQD systems despite their metallic components. Non-radiative decay pathways, including phonon-assisted relaxation and Auger recombination; compete with fluorescence emission.^48–50^ The efficiency of radiative recombination therefore depends on the balance between carrier mobility and trap-state stabilization.

Well-distributed emissive traps promote fluorescence, whereas excessive metallic conductivity can quench emission by facilitating rapid non-radiative relaxation. Photostability in MQDs is closely tied to the chemical robustness of surface terminations and defect states. Stable terminations prevent photoinduced oxidation or structural degradation, preserving emissive centers over prolonged excitation. Conversely, unstable surface chemistry can lead to fluorescence bleaching or spectral drift. Understanding excited-state dynamics in MQDs provides a unifying framework that connects quantum confinement, surface chemistry, defects, and metal electronic structure.^26,41^ This framework is essential for rational interpretation of MQD fluorescence and serves as a foundation for subsequent discussions on design strategies and sensing performance in later sections.

**Fig. 3:**
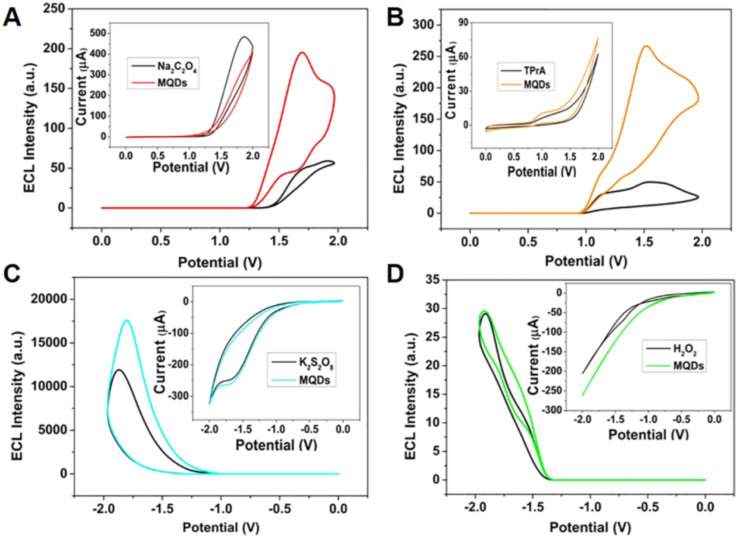
ECL–potential curves and cyclic voltammograms of MQDs in PBS (pH 7.4) containing different coreactants: (A) Na_2_C_2_O_4_, (B) TPrA, (C) K_2_S_2_O_8_, and (D) H_2_O_2_, illustrating coreactant-dependent excited-state generation, charge-transfer efficiency, and electrochemiluminescence performance. Adapted with permission from ref. 50. © 2021 American Chemical Society.

The ECL responses shown in Fig. 3 highlight how the excited-state behavior of MQDs depends strongly on the surrounding redox environment. Among the tested coreactants, potassium persulfate produces the most intense ECL signal and the lowest onset potential, indicating more efficient generation of MQDs* excited states (Fig. 3C). In contrast, weaker responses observed with sodium oxalate and TPrA, and the nearly negligible signal in the hydrogen peroxide system (Fig. 3A–D), suggest less favorable coupling between the generated radicals and MQD electronic states. These results emphasize that MQD luminescence is not governed solely by intrinsic electronic structure but also by the compatibility between MQD surface states and external redox intermediates. From a broader perspective, this behavior suggests an important guideline for MQD-based sensing systems: selecting appropriate redox partners or chemical environments is essential for maximizing excited-state population and achieving stable and amplified luminescence signals.

### 2.6. Comparison of MQDs with other quantum dot systems: distinct photophysical and structural advantages

While several classes of fluorescent quantum dots—including semiconductor quantum dots and carbon dots—have been widely explored for optical sensing, MQDs exhibit a set of structural and photophysical characteristics that distinguish them from these conventional nanomaterials. Carbon dots, for example, derive most of their fluorescence from surface states or molecular fluorophores formed during synthesis. As a result, their emission behavior is often highly sensitive to synthetic conditions and may suffer from limited electronic tenability.^18,27^ In contrast, MQDs possess a transition-metal carbide or nitride core that introduces metal d-orbitals into the electronic structure. These d-states participate directly in optical transitions, enabling hybrid metal–ligand charge-transfer processes that are largely absent in purely carbon-based nanomaterials.

Another important distinction arises from surface chemistry. Although both MQDs and carbon dots contain abundant surface functional groups, the terminations in MQDs (*e.g.*, –O, –OH, –F, –Cl) are structurally integrated into the MXene lattice rather than merely adsorbed ligands. This structural integration provides stronger electronic coupling between the surface and the core, allowing surface chemistry to modulate electronic states more efficiently. Consequently, MQDs often display stronger responsiveness to external perturbations such as ionic species, redox-active molecules, and interfacial charge transfer processes.

From an architectural perspective, MQDs also offer advantages for ratiometric sensing platforms. Their heterogeneous emissive landscape—originating from quantum confinement, metal d-state participation, surface terminations, and defect states—naturally supports multi-channel emission behavior.^36,42,48^ This intrinsic heterogeneity can be strategically harnessed to construct self-referenced sensing systems without necessarily introducing external fluorophores. In contrast, many carbon dot systems require additional dyes or secondary emitters to achieve stable ratiometric outputs.

Collectively, these features position MQDs as a distinct class of fluorescent nanomaterials that bridge metallic and semiconducting photophysics. Their metal-centered electronic structure, chemically integrated surface terminations, and inherently heterogeneous emissive states provide design flexibility that is particularly advantageous for advanced optical sensing platforms.

## 3. Architecture-level design of fluorescent and ratiometric MQD platforms

### 3.1. Single-emission *versus* ratiometric architectures: conceptual design logic and analytical implications

Fluorescent sensing platforms based on MQDs can be broadly categorized into single-emission and ratiometric architectures, each reflecting a distinct design philosophy rather than merely a difference in signal readout. In single-emission systems, fluorescence intensity or wavelength shifts of MQDs serve as the sole analytical signal. While conceptually simple, this architecture places stringent demands on signal stability, excitation reproducibility, and environmental control, as any external perturbation may be misinterpreted as an analytical response. Ratiometric architectures address these intrinsic limitations by introducing an internal reference signal that responds differently—or not at all—to external stimuli. The ratio between two emission bands, rather than an absolute intensity, becomes the analytical metric.^51–53^ In MQD-based platforms, this can be achieved through dual-emissive MQDs, hybrid assemblies with secondary fluorophores, or intrinsic emission–absorption coupling within a single nanostructure. Importantly, ratiometric design is not merely an additive modification but represents a shift toward self-calibrating sensor architectures.

From a design standpoint, the transition from single-emission to ratiometric platforms reflects a deeper understanding of MQD photophysics. The heterogeneous emissive landscape of MQDs, arising from surface states, defects, and quantum confinement, naturally lends itself to multi-channel emission. Effective ratiometric systems harness this heterogeneity deliberately rather than attempting to suppress it, converting what is often viewed as a drawback into a functional advantage. Analytically, ratiometric architectures offer enhanced resistance to fluctuations in excitation intensity, probe concentration, and optical path length. This robustness is particularly relevant for complex matrices, where signal drift is unavoidable. However, ratiometric design introduces new challenges, including spectral overlap management, emission balance, and inter-channel crosstalk, all of which must be addressed at the architectural level rather than post-measurement correction.^54–56^ Thus, the distinction between single-emission and ratiometric MQD platforms should be understood as a strategic design choice that influences signal reliability, system complexity, and long-term applicability, rather than as a simple methodological preference.

### 3.2. Engineering dual-channel emission through coupled fluorophore and hybrid nanostructures

One of the most powerful architectural approaches in MQD-based fluorescent platforms involves the deliberate coupling of MQDs with secondary emissive components to generate dual-channel optical outputs. These hybrid nanostructures are not simply physical mixtures but electronically or optically coupled systems designed to produce correlated yet distinct emission behaviors. Coupling strategies include MQD–organic dye assemblies, MQD–carbon dot hybrids, and MQD–semiconductor nanocrystal composites. In such systems, the secondary fluorophore may serve as a reference emitter, an energy donor or acceptor, or a modulator of MQD emission dynamics.^57,58^ The design challenge lies in achieving controlled interfacial interactions that enable predictable signal transduction without compromising photostability.

A key consideration in hybrid architectures is spectral compatibility. The absorption and emission profiles of MQDs must be aligned appropriately with those of the coupled fluorophore to enable efficient energy transfer or optical filtering. Improper alignment can result in signal distortion, non-linear response, or complete quenching of one emission channel. Consequently, architectural design often precedes material synthesis, reversing the traditional workflow of “material first, function later”. Another critical factor is spatial organization. The distance between MQDs and secondary fluorophores governs the efficiency of Förster resonance energy transfer or inner filter effects. Nanoscale control over spacing—achieved through molecular linkers, polymer matrices, or self-assembled frameworks—is therefore central to reproducible dual-emission behavior.^59–61^

Importantly, hybrid architectures also enable functional decoupling of signal channels. One emission band can be engineered to remain invariant under environmental changes, acting as an internal standard, while the other responds dynamically to perturbations. This decoupling significantly enhances signal fidelity without requiring complex data processing.^62,63^ Coupled-fluorophore architectures represent a shift toward modular sensor design, where MQDs act as programmable building blocks within larger optical systems rather than as isolated emitters.

### 3.3. Intrinsic ratiometric behavior enabled by surface-state and electronic heterogeneity

Beyond hybrid systems, MQDs possess an inherent capacity for ratiometric fluorescence arising from their complex electronic and surface-state heterogeneity. Unlike conventional quantum dots engineered for uniform emissive behavior, MQDs naturally host multiple emissive centers associated with basal planes, edges, defects, and surface terminations. When properly controlled, this multiplicity can give rise to intrinsic dual- or multi-emission behavior without external fluorophores. Intrinsic ratiometric architectures rely on the differential sensitivity of distinct emissive states to external perturbations. For example, surface-state emission may respond strongly to local electronic changes, while core-related emission remains relatively stable. The ratio between these emissions thus encodes environmental information in a self-referenced manner.^64–66^

Designing such intrinsic ratiometric systems requires precise control over MQD synthesis parameters, including size distribution, termination chemistry, and defect density. Rather than minimizing heterogeneity, architectural design aims to stabilize specific emissive populations while maintaining sufficient contrast between them. This approach marks a conceptual departure from traditional nanomaterial synthesis paradigms that prioritize uniformity above all else.

Another advantage of intrinsic ratiometric MQDs is architectural simplicity. Eliminating secondary fluorophores reduces synthetic complexity, potential leaching issues, and long-term instability. However, intrinsic systems demand rigorous photophysical characterization to ensure that emission ratios are reproducible and interpretable across different batches.^67–70^ From a platform perspective, intrinsic ratiometric behavior positions MQDs as self-contained optical transducers. This capability is particularly attractive for applications requiring miniaturization, integration into solid substrates, or operation under variable excitation conditions.

### 3.4. Platform robustness through signal normalization, self-calibration, and optical redundancy

A defining feature of advanced MQD-based fluorescent platforms is the incorporation of architectural elements that enhance robustness beyond raw sensitivity. Signal normalization, self-calibration, and optical redundancy are increasingly recognized as essential design principles, particularly for systems intended for real-world analytical environments. Ratiometric architectures inherently perform signal normalization by referencing one emission channel against another.^70,71^ However, robustness can be further enhanced through redundancy, where multiple independent optical pathways encode similar information. For instance, combining intensity-based and wavelength-based readouts within the same MQD platform provides cross-validation of analytical responses.

Self-calibration mechanisms can also be embedded at the architectural level. These include fixed reference emissions, excitation-independent ratios, or built-in optical standards that compensate for photobleaching and instrumental drift. Such features reduce reliance on external calibration protocols and improve reproducibility across measurement sessions. Importantly, robustness-oriented design does not necessarily increase system complexity. In many cases, it involves reinterpreting existing MQD photophysical features—such as excitation-dependent emission or multi-state fluorescence—as functional assets rather than sources of uncertainty.^29,72^

From an editorial perspective, platforms emphasizing robustness align closely with current expectations for translational relevance, even in fundamentally oriented review articles. They demonstrate awareness of analytical realities without prematurely entering application-specific discussions. Architecture-level design of MQD fluorescent platforms represents a maturation of the field, shifting emphasis from material novelty toward signal integrity, reliability, and system-level thinking.^55,73^ This conceptual evolution provides a natural bridge between fundamental photophysics and application-focused analyses addressed in subsequent sections.

Table 1 summarizes key architecture-level design dimensions that govern the performance of fluorescent and ratiometric MQD platforms, emphasizing system-level considerations rather than material novelty. Each design dimension reflects a strategic decision that influences signal reliability, robustness, and interpretability. Importantly, the table illustrates how ratiometric behavior can emerge either intrinsically from MQD electronic heterogeneity or extrinsically through hybrid architectures incorporating secondary fluorophores. A central insight highlighted by this comparison is the trade-off between architectural simplicity and signal stability. While single-emission systems offer straightforward implementation, they remain inherently vulnerable to environmental and instrumental fluctuations. In contrast, dual-emission and reference-channel designs embed normalization directly into the platform, enhancing analytical reliability at the cost of increased structural and spectral complexity. The table also underscores the importance of spatial and spectral engineering in achieving predictable optical behavior. Nanoscale control over emitter spacing and band alignment is not merely a synthetic detail but a determinant of whether coupled systems function as stable ratiometric platforms or unstable intensity-modulated probes. Furthermore, the use of surface-state and defect-derived emission as functional signal channels represents a conceptual shift, transforming MQD heterogeneity into an asset rather than a limitation.

**Table 1:** Architecture-level design elements governing fluorescent and ratiometric MQD platforms

Design dimension	Structural basis	Optical consequence	Analytical advantage	Key design trade-off
Emission mode	Single emissive MQDs	Intensity-based signal	Simplicity of readout	Sensitivity to external fluctuations
Dual-emission strategy	Intrinsic MQD heterogeneity	Two correlated emission bands	Built-in signal referencing	Emission overlap management
Hybrid architecture	MQD–fluorophore coupling	Coupled or decoupled dual emission	Enhanced signal stability	Interfacial control complexity
Spatial organization	Nanoscale separation control	Tunable energy transfer efficiency	Predictable ratiometric behavior	Distance-dependent signal drift
Spectral engineering	Band alignment design	Controlled Stokes shift	Reduced background interference	Limited material combinations
Reference channel design	Invariant emission center	Excitation-independent ratio	Self-calibration capability	Reduced dynamic range
Signal redundancy	Multi-path optical outputs	Cross-validated response	Increased robustness	Increased data interpretation load
Surface-state utilization	Engineered emissive traps	Differential sensitivity	Intrinsic ratiometric response	Batch-to-batch variability
Platform integration	Composite or solid-state assembly	Preserved emission contrast	Improved operational stability	Fabrication scalability limits

## 4. Performance-driven applications of MQD-based fluorescent and ratiometric sensors in food safety analysis

### 4.1. Ratiometric fluorescent detection of biogenic amines in food

MQDs have emerged as robust fluorescent platforms for the detection of biogenic amines, particularly histamine, which poses significant health risks in spoiled and processed food products and is a primary quality/safety marker in fish, meat, and fermented foods. The integration of ratiometric design strategies with molecular imprinting layers allows for both high sensitivity and visual detectability. For instance, blue/orange MQDs functionalized with molecularly imprinted polymers exhibit a dual-emission profile, where the blue emission serves as the analyte-responsive signal and the orange emission functions as an internal reference. This dual-emission approach significantly reduces background interference, enabling a linear fluorescence response over histamine concentrations ranging from 1 to 60 µM with limits of detection as low as 21.9 nM for fluorescence-based sensing and 92.2 nM for visual detection.^74,75^

A critical aspect contributing to high analytical performance is the design of MQDs with tailored surface chemistry. Ethylenediamine-functionalized Ti_3_C_2_ MQDs (EDA-MQDs) exploit chelation and inner filter effects to achieve sequential detection of Fe^3+^ and histamine. In this system, the presence of Fe^3+^ quenches MQD fluorescence, whereas subsequent binding of histamine restores the emission, generating an off–on response. Such sequential dual-analyte detection enables simultaneous monitoring of metal ions and biogenic amines directly in food matrices (*e.g.*, canned fish and meat products) without compromising specificity, thus addressing realistic quality control requirements in food supply chains. Performance validation in real food samples demonstrated excellent recovery rates and reproducibility, highlighting the practical applicability of these sensors in point-of-use scenarios.^76^

In addition, the ratiometric nature of these platforms provides inherent self-calibration, mitigating the effects of variations in excitation intensity, sample turbidity, or environmental pH. This is particularly important for histamine detection in complex matrices such as fish, meat, and fermented products, where interference from endogenous components can otherwise compromise sensor reliability. Overall, ratiometric MQD sensors demonstrate superior analytical figures of merit compared to traditional single-emission probes, including higher sensitivity, broader linear range, visual detectability, and robustness across diverse food matrices, making them well suited for routine histamine monitoring in real food analysis and food safety control.

### 4.2. Antibiotic and tetracycline sensing using doped MQDs

The detection of antibiotic residues in food, especially tetracycline (TC) and chloramphenicol (CPL), is a critical application of MQDs-based fluorescent sensors. Nitrogen- and boron-doped Ti_3_C_2_ MQDs, combined with europium ions (Eu^3+^), constitute an effective ratiometric fluorescence platform for tetracycline detection. Upon addition of TC, the blue fluorescence emission of MQDs is quenched, while red emission from Eu^3+^ is enhanced due to synergistic antenna and inner filter effects. This dual-emission behavior enables point-of-care testing (POCT) with rapid colorimetric readouts, which can be interpreted *via* smartphone-based applications.^77^ The platform demonstrated a detection limit of 20 nM and successful application in milk samples, confirming its practical utility for food safety monitoring and regulatory screening of antibiotic residues in dairy products.

Similarly, highly photostable N/S-co-doped Nb_2_C MQDs allow quantitative detection of CPL *via* a “turn-off” fluorescence mechanism. The interaction between Nb_2_C-MX QDs and CPL forms non-fluorescent complexes, effectively quenching emission with a linear response over 20–240 µM and a detection limit of 76.3 nM. The sensor maintained exceptional stability under UV irradiation, variable pH, and ionic conditions, while interference studies confirmed selectivity against amino acids and other biomolecules. Recovery tests in water, milk, and human biological samples ranged from 92.31% to 98.24%, demonstrating robust analytical performance and underscoring that the sensor can reliably quantify chloramphenicol residues in relevant food matrices as well as in exposure-related biological samples.^78^

A key advantage of doped MXene QDs in antibiotic sensing is their tunable emission characteristics combined with high surface functionality, allowing selective binding and energy transfer interactions that enhance sensitivity. Additionally, the ratiometric output provides reliable internal referencing to correct for environmental or instrumental variability. Collectively, these platforms showcase the translation of nanoscale material design into actionable, quantitative food safety monitoring tools with reproducible, high-performance metrics specifically tailored for routine antibiotic residue analysis in foods of animal origin.

### 4.3. Dual-modal nitrite detection using functionalized MQDs

Nitrite (NO_2_^−^) is a widely monitored food additive due to its potential toxicity at elevated concentrations. Dual-modal detection strategies using MQDs allow simultaneous fluorescence and colorimetric readouts for rapid, real-time sensing. Microwave-assisted N,P-doped Ti_3_C_2_ MQDs serve as green-emitting probes, whose fluorescence is quenched by 1,10-phenanthroline–Fe(ii) complexes through inner filter effects. Upon addition of nitrite, redox reactions restore fluorescence and induce a gradual color transition from orange to colorless, enabling direct visual interpretation of analyte concentration. This dual-modality enhances reliability by cross-validating results through optical and visual channels, particularly in complex food matrices such as cured meats and processed products in which nitrite levels must be tightly controlled.^79^

Fluorescence resonance energy transfer (FRET) between glutathione-functionalized Ti_3_C_2_ MQDs and oxidation products of *o*-phenylenediamine allows “naked-eye” colorimetric ratiometric detection of uric acid as a biomarker for food spoilage. The MQD emission at 430 nm is quenched while the oxOPD emission at 568 nm increases proportionally with analyte concentration. The visible color change provides an immediate qualitative assessment, while ratiometric fluorescence ensures quantitative evaluation. Such designs leverage both the intrinsic optical properties of MQDs and analyte-specific enzymatic transformations, demonstrating a versatile framework for dual-channel sensing of spoilage-related biomarkers in perishable foods.^80^

These dual-modal MQD sensors exemplify the integration of surface-functionalized nanomaterials with chemical recognition elements to achieve rapid, sensitive, and reliable detection. The combination of ratiometric fluorescence with visual readouts enhances both analytical precision and user accessibility, making these systems particularly suitable for on-site monitoring and consumer-facing food safety applications, for example rapid screening of nitrite in cured meats or uric-acid-related spoilage indicators in high-protein food products. Importantly, these designs prioritize operational simplicity without compromising quantitative rigor, a critical requirement for practical deployment in food industry workflows.

**Fig. 4:**
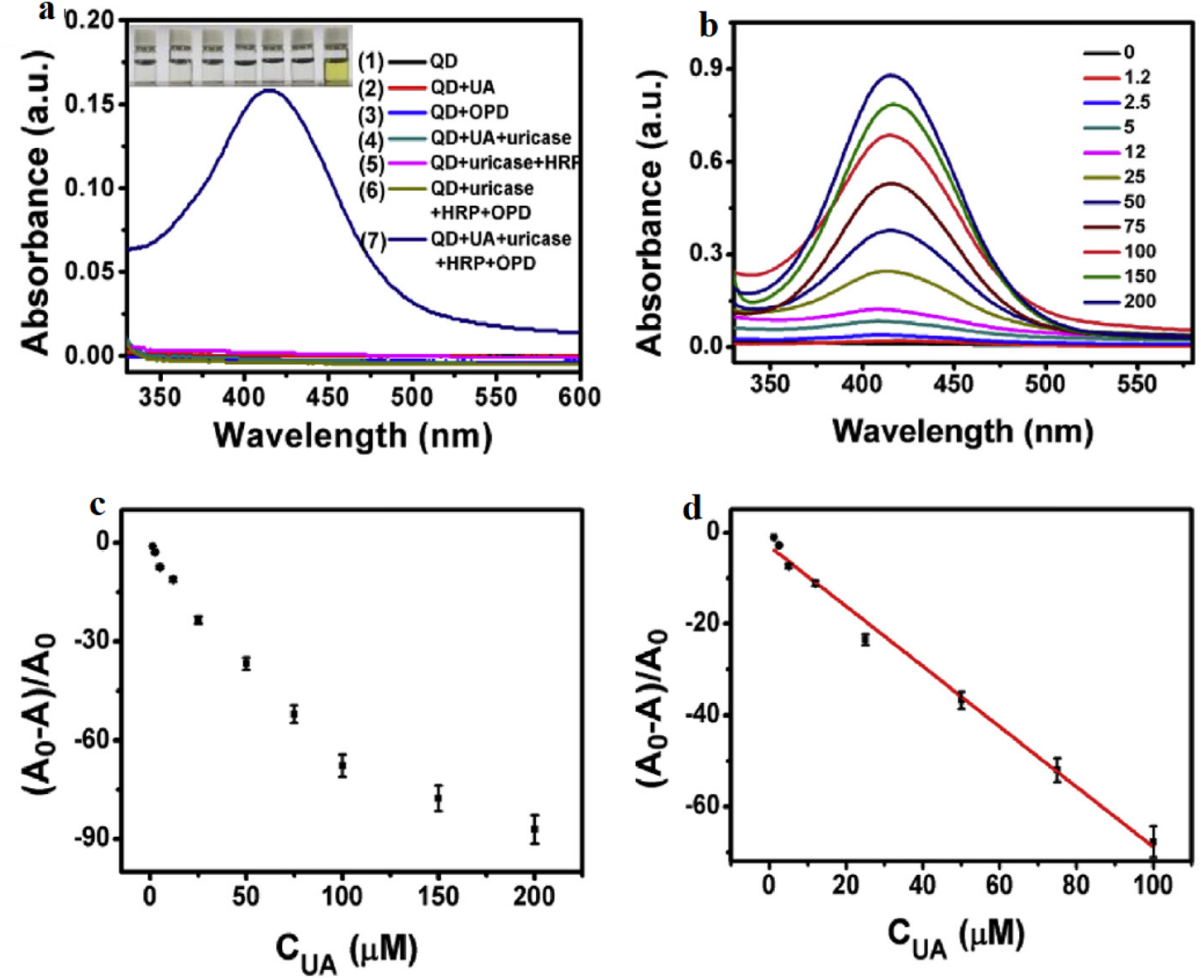
(a) Absorption spectra of GSH–Ti_3_C_2_ MQDs incubated with individual interferents and the complete UA/uricase/HRP/OPD system. (b) Absorbance evolution with increasing UA (0–200 µM). (c) Scatter plot of (*A*_0_ − *A*)/*A*_0_*versus* UA concentration. (d) Linear calibration curve (1.2–100 µM) for quantitative determination; error bars represent triplicate measurements. Adapted with permission from ref. 80. © 2020 Elsevier B.V.

Panels (4a–4d) illustrate the analytical principle and quantitative performance of the GSH–Ti_3_C_2_ MQD-based dual-modal sensing platform for uric acid detection. As shown in Fig. 4a, negligible spectral variation is observed when the MQDs are exposed to individual interfering species, whereas the complete UA/uricase/HRP/OPD cascade produces a distinct absorption band at 425 nm associated with oxOPD formation. This behavior highlights that signal generation is governed primarily by the enzymatic oxidation pathway rather than nonspecific interactions, demonstrating the role of biochemical selectivity in stabilizing MQD-based optical responses. With increasing UA concentration (Fig. 4b), the progressive growth of the 425 nm absorption band reflects enhanced oxOPD production, which simultaneously modulates MQD emission through FRET interactions. The concentration-dependent evolution summarized in Fig. 4c confirms the systematic optical response of the hybrid system. A well-defined linear relationship within the 1.2–100 µM range (Fig. 4d) further demonstrates the quantitative capability of the platform, yielding a detection limit of 200 nM. Beyond the individual measurements, these results illustrate a broader sensing principle: coupling enzymatic specificity with MQD-mediated optical transduction enables highly selective and sensitive detection while maintaining compatibility with complex food matrices.

### 4.4. Smartphone-integrated ratiometric sensing for *in situ* food monitoring

The integration of MQD platforms with smartphone-based detection systems has emerged as a transformative approach for real-time food safety monitoring directly at points of production, storage, distribution, and retail. Ratiometric fluorescent sensors utilizing blue/orange MQDs encapsulated in molecularly imprinted polymers enable simultaneous quantitative measurement and visual readout *via* smartphone cameras. The dual-emission mechanism allows calibration-free analysis, where the ratio of blue to orange fluorescence directly correlates with analyte concentration, such as histamine in fish or meat products. Analytical performance studies show linear detection ranges of 1–60 µM with limits of detection as low as 21.9 nM, demonstrating sensitivity suitable for regulatory compliance.

Smartphone integration leverages built-in optical sensors and computational algorithms to process colorimetric or ratiometric signals in real time, providing rapid feedback without requiring complex laboratory instrumentation. The portability and accessibility of these platforms make them particularly suitable for field applications, catering to food producers, distributors, retailers, and consumers alike by enabling *in situ* assessment of histamine levels in fish and meat products along the entire food supply chain.^74,75^ Additionally, the combination of molecular imprinting and MQD functionalization enhances selectivity, mitigating interference from coexisting ions or biomolecules commonly present in food matrices.

In terms of performance validation, *in situ* detection experiments using real food samples achieved recoveries between 96.52% and 105.32%, confirming both the robustness and practicality of smartphone-assisted MQD sensors. The ability to simultaneously provide quantitative and visual outputs enhances the operational flexibility of these sensors, bridging the gap between laboratory precision and field applicability. By transforming MQDs into smart, user-friendly devices, this approach exemplifies the potential of nanomaterial-enabled ratiometric sensing to revolutionize real-time food safety monitoring.

### 4.5. Multi-analyte detection *via* functionalized MQDs

Simultaneous detection of multiple food contaminants represents a critical advancement in analytical food safety, and functionalized MQDs provide a versatile platform to address this challenge with high sensitivity, selectivity, and throughput. Ethylenediamine-functionalized Ti_3_C_2_ MQDs (EDA-MQDs) exemplify the potential of sequential off–on fluorescence mechanisms for dual analyte detection, specifically Fe^3+^ and histamine. In this system, fluorescence quenching occurs *via* the inner filter effect induced by Fe^3+^, while the chelation of histamine with Fe^3+^ modulates the free ion concentration, leading to fluorescence recovery. This dual-analyte sensor achieves limits of detection as low as 0.11 µM for Fe^3+^ and 46 nM for histamine, with linear detection ranges of 1–100 µM and 1–60 µM, respectively, demonstrating both high sensitivity and practical applicability in complex food matrices such as canned fish and meat products, where simultaneous control of metal ion contamination and biogenic amine accumulation is required.^76^ The selectivity and reproducibility of the EDA-MQDs platform ensure minimal interference from coexisting ions and biomolecules, supporting its deployment for integrated food monitoring.

Doped Ti_3_C_2_ MQDs expand the multi-analyte detection capability through energy transfer-based ratiometric designs. Nitrogen/boron co-doped MQDs, coupled with Eu^3+^ ions, enable dual-emission detection of tetracycline, where quenching of MQD fluorescence is precisely correlated with target concentration, while the Eu^3+^ emission serves as a stable internal reference.^77^ Similarly, nitrogen/phosphorus-doped MQDs paired with 1,10-phenanthroline–Fe^2+^ complexes provide a dual-modal readout for nitrite, combining fluorescence and colorimetric signals. Such designs exploit the tunable optical and electronic properties of MQDs to enable selective response without crosstalk between analytes.^79^

These advanced functionalization strategies allow the construction of MQD-based hybrid nanoplatforms capable of addressing diverse contamination challenges in food systems. Multi-analyte detection reduces assay time and operational cost, while facilitating comprehensive screening in a single measurement, thereby enhancing throughput and practical relevance. Furthermore, the integration of ratiometric sensing, dual emission and dual-modal readouts increases robustness against environmental variability and matrix interference, critical for field-deployable and regulatory-compliant applications. Collectively, these approaches underscore the adaptability of functionalized MXene QDs in enabling high-precision, rapid, and multiplexed food safety analysis, positioning them as a next-generation tool for real-time monitoring of chemical contaminants and biogenic amines in complex food matrices, with direct relevance to hazard control and quality assurance in modern food analysis workflows.^76,79^

**Fig. 5:**
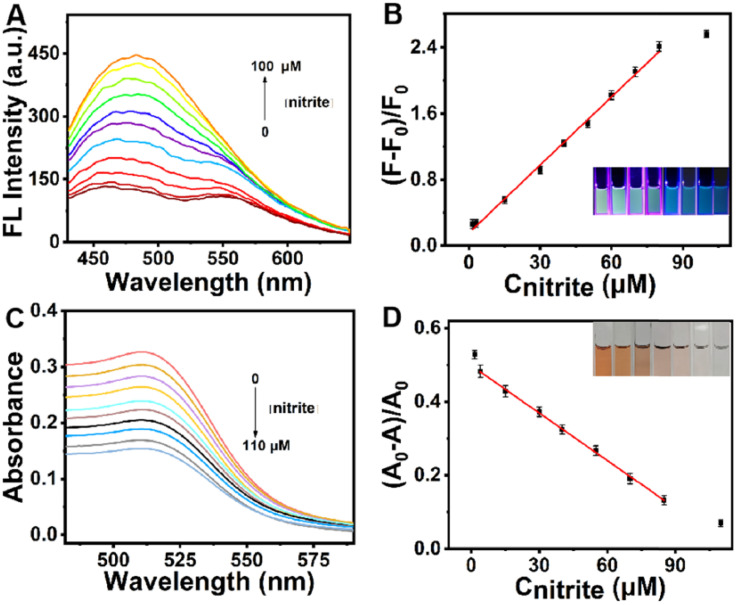
(A) Fluorescence emission spectra of the N,P-Ti_3_C_2_ MQDs/Phen/Fe^2+^ sensing system upon addition of NO_2_^−^ (0–100 µM). (B) Linear relationship between (*F* − *F*_0_)/*F*_0_ and NO_2_^−^ concentration (1.5–80 µM); inset: UV photographs under 395 nm excitation. (C) UV-vis absorption spectra of the sensing platform with increasing NO_2_^−^ (0–110 µM). (D) Linear plot of (*A*_0_ − *A*)/*A*_0_*versus* NO_2_^−^ concentration (4–85 µM); inset: corresponding daylight images showing colorimetric response. Adapted with permission from ref. 79. © 2022 Elsevier B.V.

Panels (5A–5D) demonstrate the dual-emission and dual-modal sensing behavior of the N,P-Ti_3_C_2_ MQDs/Phen/Fe^2+^ platform for nitrite detection. As illustrated in Fig. 5A, the initially quenched fluorescence of the MQDs–Phen/Fe^2+^ complex progressively recovers with increasing NO_2_^−^ concentration, indicating that nitrite perturbs the coordination environment of the Fe^2+^–phenanthroline complex and consequently alters the photoinduced electron or energy transfer processes within the hybrid system. The linear relationship between fluorescence recovery ((*F* − *F*_0_)/*F*_0_) and nitrite concentration over 1.5–80 µM (Fig. 5B) demonstrates the capability of MQD-based ratiometric fluorescence to provide sensitive quantitative detection, with a calculated detection limit of 0.25 µM. Complementary colorimetric behavior is observed in Fig. 5C, where the characteristic absorption band of the MQDs/Phen/Fe^2+^ complex gradually decreases upon nitrite addition, reflecting structural or coordination changes within the chromophoric system. The corresponding linear relationship between ((*A*_0_ − *A*)/*A*_0_) and nitrite concentration (Fig. 5D) confirms the reliability of the absorbance channel, while the visible color transition offers an intuitive visual readout. Taken together, these results highlight a broader design concept for MQD-based sensors: integrating independent yet correlated fluorescence and colorimetric outputs significantly improves analytical robustness, reduces susceptibility to matrix interference, and enhances suitability for on-site food safety monitoring.

### 4.6. Visual and quantitative colorimetric–fluorometric readouts for practical food analysis

Combining colorimetric and ratiometric fluorescence responses in MQD-based sensors enables practical, user-friendly detection of key food safety markers, including histamine, nitrite, and uric acid, directly in real food samples such as fish, meat products, and fermented foods. The N/P-doped Ti_3_C_2_ MQD/Phen–Fe^2+^ system, for instance, exhibits dual-channel readout: fluorescence quenching in the green emission band is complemented by a visually perceptible color change from orange to colorless upon nitrite exposure.^79^ This dual-modal design facilitates both quantitative laboratory analysis and immediate on-site assessment, allowing rapid decisions regarding food safety.

“Naked-eye” detection platforms using GSH-functionalized Ti_3_C_2_ MQDs exploit FRET-based ratiometric emission between MQDs and enzymatically oxidized analytes (*e.g.*, oxOPD from uric acid). Increasing analyte concentration systematically quenches MQD emission while enhancing the emission of the oxidation product, providing a colorimetric shift detectable by the eye and a quantitative fluorescence signal for rigorous analysis. Detection limits in such systems are in the nanomolar range, with recoveries validated in biological fluids and food matrices, confirming both sensitivity and practical applicability.^80^

These systems illustrate the synergy between nanomaterial optical tunability and simple visual outputs. The dual-readout capability is particularly important in food safety contexts, where rapid, reliable detection is required for real-time monitoring, consumer safety, and regulatory compliance. By integrating both quantitative and qualitative signals into a single assay, MQD-based sensors deliver multifunctional performance with minimal operational complexity, positioning them as ideal candidates for future smart food analysis technologies.

### 4.7. Comparative framework and design principles in MQD-based food sensors

Recent progress in MXene quantum dots (MQDs) for food safety analysis highlights the importance of a comparative, principles-based framework connecting photophysics, sensor architecture, and analytical performance. Unlike prior narrative reports, comparative assessment across representative systems (Table 1) reveals that analytical success originates from two convergent design dimensions: (i) rational compositional engineering and (ii) architecture-performance coupling.

From a composition standpoint, heteroatom doping (*e.g.*, N, B, P, S) and molecular functionalization control MQDs' band structure and excitonic behavior, thereby dictating sensing mechanism and selectivity. N/B-co-doped Ti_3_C_2_ MQDs integrated with Eu^3+^ ions^77^ optimize antenna-assisted energy transfer for red-shifted ratiometric emission, achieving low-nanomolar LODs for tetracycline. Similarly, N/P-doped Ti_3_C_2_ MQDs in Fe^2+^–phenanthroline systems^79^ employ inner-filter modulation to deliver dual-modal nitrite detection with sub-micromolar precision, whereas EDA-functionalized MQDs^76^ exploit specific Fe^3+^ chelation to enable sequential Fe^3+^/histamine analysis. These examples collectively demonstrate that tailored surface chemistry governs photophysical coupling, establishing predictable structure–function correlations fundamental for design optimization.

Architecturally, systems integrating ratiometric or dual-modal outputs consistently outperform single-emission probes, offering self-referencing stability against optical noise and matrix interference. The smartphone-readout histamine sensor^74,75^ exemplifies the transition from lab prototypes to field-deployable devices, providing real-time quantification with recoveries >95%. Comparatively, multi-analyte EDA-MQDs and doped MQD/lanthanide hybrids^76,77,79^ deliver extended linear ranges and multiplexed detection without signal crosstalk, emphasizing the scalability of modular hybridization strategies.

Overall, this comparative analysis suggests three overarching design rules:

(1) Electronic structure modulation through doping or edge functionalization determines sensitivity and selectivity;

(2) Optical redundancy *via* ratiometric or dual-modal architecture ensures reliability across dynamic food matrices;

(3) Integration with portable or digital interfaces—such as smartphone or paper-supported assays—bridges fundamental photophysics with practical analytics.

By establishing these cross-study relationships, the review advances beyond descriptive summarization toward a system-level synthesis that operationalizes the interplay between composition, architecture, and performance metrics for MQD-based food sensors.

### 4.8. Systematic comparative analysis of representative MQD-based food sensors

To further operationalize the link between MQD photophysics, sensing architecture, and analytical performance, a systematic comparison of representative sensing systems is summarized in Table 1. Evaluating these platforms across compositional design, sensing mechanisms, and analytical metrics reveals clear trends that help rationalize the performance advantages of MQD-based sensors in food safety analysis.

One consistent observation across studies is the decisive role of surface engineering and heteroatom doping in controlling MQD optical behavior and sensing selectivity. For instance, N,B-co-doped Ti_3_C_2_ MQDs combined with Eu^3+^ ions form a ratiometric fluorescence platform capable of detecting tetracycline residues in milk with a detection limit of 20 nM.^77^ In this system, the MQDs act as an energy donor while Eu^3+^ serves as a stable reference emitter, enabling self-calibrated quantification even in optically complex food matrices. Similarly, N,P-doped Ti_3_C_2_ MQDs incorporated into Fe^2+^–phenanthroline complexes enable dual-modal detection of nitrite through coupled fluorescence and colorimetric responses, achieving detection limits of 0.25 µM while providing both quantitative and visual outputs suitable for on-site monitoring.^79^

Functional group modification provides another pathway to tailor sensor selectivity and multi-analyte capability. Ethylenediamine-functionalized Ti_3_C_2_ MQDs (EDA-MQDs) demonstrate sequential fluorescence responses toward Fe^3+^ and histamine, exploiting metal–ligand coordination and inner-filter effects to enable dual-target detection in food matrices.^76^ The platform achieves detection limits as low as 0.11 µM for Fe^3+^ and 46 nM for histamine, highlighting how specific ligand–analyte interactions can be harnessed to construct multiplexed sensing systems without compromising analytical precision.

Beyond compositional design, the choice of sensing mechanism strongly influences analytical performance. Systems relying on fluorescence resonance energy transfer (FRET), inner filter effects (IFE), or antenna-mediated energy transfer tend to offer improved sensitivity compared with simple fluorescence quenching approaches. For example, glutathione-functionalized Ti_3_C_2_ MQDs coupled with enzymatic oxidation of *o*-phenylenediamine enable ratiometric detection of uric acid through FRET interactions between MQD emission and oxOPD fluorescence, providing a detection limit of 200 nM with reliable performance in food and biological matrices.^80^ In contrast, sensors based solely on static quenching mechanisms, such as N/S-co-doped Nb_2_C MQDs for chloramphenicol detection, typically display broader linear ranges but slightly higher detection limits.^78^

Finally, the comparative dataset highlights the increasing emphasis on practical deployment and user accessibility. Platforms integrating smartphone-assisted readout or naked-eye colorimetric responses—such as the dual-emission histamine sensor using molecularly imprinted MQDs—demonstrate strong recoveries in real food samples while enabling portable, calibration-free analysis.^74,75^ Collectively, the comparison underscores that optimal MQD sensing systems arise from the synergistic combination of rational material engineering, multi-channel optical transduction, and application-oriented device integration, providing a clear roadmap for the development of next-generation food safety sensors.

Table 2 provides a comprehensive comparative overview of MQDs-based fluorescent and ratiometric sensors for food safety analysis. The table illustrates how diverse functionalization strategies, including molecular imprinting, N/B or N/P co-doping, and coordination with metal ions, significantly enhance the selectivity, sensitivity, and photostability of MQDs. Blue/orange dual-emission designs enable ratiometric fluorescence, minimizing environmental interference and providing self-calibration, whereas visual and smartphone-assisted readouts offer practical, on-site monitoring capabilities. The detection ranges, spanning nanomolar to micromolar concentrations, demonstrate that MQDs can sensitively respond to critical analytes such as histamine, Fe^3+^, tetracycline, nitrite, chloramphenicol, and uric acid. Recovery studies in real samples, including fish, meat, milk, and biological fluids, confirm both reliability and applicability. Furthermore, the integration of inner filter effects, antenna effects, and chelation interactions underlines the sophisticated mechanisms exploited for target recognition. Collectively, this table highlights the versatility of MQDs as tunable nanomaterials capable of delivering high-performance, portable, and rapid sensing platforms for advanced food safety and point-of-care applications, paving the way for next-generation analytical technologies.

**Table 2:** Performance summary of MQDs-based fluorescent and ratiometric sensors for food safety analysis

MQD type/functionalization	Target analyte	Detection range	LOD	Real sample application	Ref.
Blue/orange MQDs + MIP	Histamine	1–60 µM	21.9 nM (fluorescence), 92.2 nM (visual)	Fish and meat samples; recoveries 96.52–105.32%	74
EDA-functionalized Ti_3_C_2_ MQDs	Fe^3+^ & histamine	1–100 µM (Fe^3+^), 1–60 µM (histamine)	0.11 µM (Fe^3+^), 46 nM (histamine)	Real food matrices; selective dual-detection	76
N,B-Ti_3_C_2_ MQDs + Eu^3+^	Tetracycline	Not explicitly stated	20 nM	Milk samples; POCT paper test	77
N,P-Ti_3_C_2_ MQDs + Phen–Fe^2+^	Nitrite	Not explicitly stated	Not explicitly stated	Paper-based strips; smartphone-assisted real-time detection	79
N,S-Nb_2_C MQDs	Chloramphenicol	20–240 µM	76.3 nM	Tap water, milk, human blood/urine; recoveries 92.31–98.24%	78
Blue/orange MQDs + BMQDs@MIPs	Histamine	1–60 µM	21.9 nM (fluorescence), 92.2 nM (visual)	Fish and meat samples; recoveries 96.52–105.32%	75
GSH-Ti_3_C_2_ MQDs + OPD/HRP	Uric acid	Not explicitly stated	Not explicitly stated	Biological fluids; visual and ratiometric fluorescence detection	80

### 4.9. Toxicity, environmental impact, and biocompatibility considerations of MQD-based sensors in food analysis

While MQDs demonstrate excellent analytical performance in fluorescence-based sensing, their practical application in food safety monitoring also requires careful consideration of toxicity, environmental impact, and regulatory safety aspects. Because these sensors may interact with food matrices or be used in portable monitoring devices, understanding their biocompatibility and environmental footprint is essential for responsible technological deployment.

Compared with conventional semiconductor quantum dots such as CdSe or PbS, MQDs are generally considered less hazardous because they do not rely on highly toxic heavy metals. However, their biocompatibility depends strongly on factors such as surface termination, particle size, oxidation state, and synthesis conditions. MXene-derived nanomaterials often contain surface functional groups including –OH, –O, and –F, which can influence both colloidal stability and biological interactions.^73–76^ Although several studies suggest relatively low cytotoxicity of Ti_3_C_2_-derived MQDs at typical sensing concentrations, systematic toxicological evaluations remain limited, particularly under long-term exposure or environmentally relevant conditions.

In comparison, carbon dots (CDs) are widely recognized for their excellent biocompatibility, low toxicity, and environmentally benign composition, which has contributed to their extensive use in biosensing and bioimaging applications. As a result, CDs are often considered a benchmark for safe fluorescent nanomaterials. MQDs, while offering distinct advantages in terms of electrical conductivity, tunable photophysical behavior, and rich surface chemistry, must therefore be evaluated against such established systems to ensure comparable safety profiles.

From a regulatory perspective, materials intended for food-related applications must comply with strict safety standards regarding nanoparticle migration, environmental persistence, and potential human exposure. Strategies such as polymer encapsulation, immobilization on solid substrates, or integration into paper-based analytical devices can significantly reduce the risk of nanoparticle release while maintaining sensing performance.^78–80^

Overall, future development of MQD-based food sensors should balance analytical performance with comprehensive safety assessment. Integrating toxicity studies, environmental impact evaluation, and safer-by-design material engineering will be essential to facilitate regulatory acceptance and ensure sustainable implementation of MQD technologies in food safety monitoring.

## 5. From laboratory prototypes to food safety practice: challenges, standardization, and translational perspectives

### 5.1. Scalability and reproducibility of MQD sensors

The synthesis of MQDs has advanced significantly in laboratory settings, enabling controlled size distribution, surface terminations, and functionalization for selective analyte detection. Methods such as hydrothermal, ultrasonic-assisted, and microwave-assisted syntheses provide reproducible optical properties and high quantum yields at a small scale. However, the transition from milligram to gram or kilogram production introduces critical challenges in maintaining uniformity and batch-to-batch reproducibility. Parameters such as reaction time, temperature gradients, precursor concentrations, and functionalization efficiency must be tightly controlled to avoid variations in fluorescence intensity, emission wavelength, or stability. In addition, functionalization with molecularly imprinted polymers, metal dopants, or surface ligands adds layers of complexity that may affect the robustness of multi-analyte detection platforms.^74,76^ Ensuring consistent performance in scaled-up synthesis is essential for their practical adoption in food safety monitoring.

Scalability challenges are further compounded by the necessity for high photostability, resistance to aggregation, and long-term shelf-life in complex food matrices. MQDs intended for commercial deployment must maintain optical integrity under variations in pH, ionic strength, temperature, and exposure to light. Strategies such as surface passivation, polymer encapsulation, and post-synthetic purification can enhance stability, but each step introduces potential sources of variability. Automation and continuous-flow reactors are emerging as viable approaches to minimize human-induced variability, enabling precise control over reaction kinetics, particle size distribution, and functional group density. These approaches support the production of uniform MQD batches with predictable fluorescence responses, a prerequisite for consistent analytical performance.^78,80^

The reproducibility of multi-analyte and ratiometric MQD sensors is particularly sensitive to synthesis parameters. Functionalization strategies must ensure selective recognition without crosstalk between channels, which is critical when monitoring multiple contaminants such as biogenic amines, metal ions, or antibiotics. Quantitative evaluation through inter-batch comparisons and rigorous characterization—including fluorescence quantum yield, lifetime, and spectral stability—provides confidence in sensor reliability. Addressing these scalability and reproducibility challenges not only facilitates high-throughput production but also bridges the gap between proof-of-concept demonstrations and real-world application, ensuring that MQD-based sensors can be deployed as reliable tools in food safety monitoring at industrial and regulatory scales.^74,80^

### 5.2. Standardization and regulatory integration

For MQD sensors to gain acceptance in food safety monitoring, adherence to regulatory and standardization requirements is crucial. Agencies such as ISO, AOAC, and EFSA demand thorough validation of analytical performance, including limits of detection, linear dynamic range, selectivity, and reproducibility in real food matrices. While laboratory studies demonstrate excellent detection limits for biogenic amines, antibiotics, and metal ions, discrepancies in sample preparation, spiking protocols, and calibration procedures can hinder cross-study comparability. Standardized protocols for sensor fabrication, analyte spiking, recovery assays, and fluorescence calibration are essential for regulatory acceptance, ensuring consistency of measurements across different laboratories and operational conditions.

Beyond performance metrics, regulatory integration requires addressing biosafety and environmental concerns associated with MQDs. Potential leaching of metal species or surface ligands, accumulation in food, and environmental disposal must be systematically evaluated. Risk assessments, including cytotoxicity and bioaccumulation studies, are critical to establishing safety thresholds. Additionally, the incorporation of portable detection platforms such as smartphone-based readers introduces additional requirements for data traceability, device calibration, and user safety.^77,78^ Harmonization of reporting formats and adoption of inter-laboratory proficiency tests strengthen confidence in MQD-based sensing technologies.

Adherence to standardized guidelines enables MQD sensors to transition from experimental prototypes to tools with regulatory recognition. Validation across diverse food matrices, including milk, meat, and processed products, provides evidence for real-world applicability. Establishing benchmark analytical protocols, integrating quality control metrics, and documenting sensor performance according to regulatory frameworks ensure reproducibility and comparability.^79,80^ These measures accelerate translational adoption, positioning MQD-based sensors as credible, field-ready solutions for rapid, sensitive, and reliable food safety analysis, bridging the gap between laboratory innovation and consumer protection.

### 5.3. Translational perspectives and field deployment

The translation of MQD sensors from laboratory prototypes to practical food safety applications requires careful consideration of field-deployment challenges. Their high sensitivity, tunable photoluminescence, and ratiometric capabilities make them ideal candidates for real-time monitoring of contaminants, including biogenic amines, antibiotics, and heavy metals. However, practical deployment demands device integration strategies that preserve optical and chemical performance under diverse environmental conditions. Factors such as temperature fluctuations, pH variations, and potential interference from complex food matrices can significantly impact the reliability of fluorescence-based readouts.

Integration into portable platforms, including microfluidic chips, lateral flow devices, and smartphone-assisted readers, is essential for point-of-use applications. Encapsulation of MQDs within polymer matrices or hydrogel carriers can enhance stability while maintaining analyte accessibility.^76,78^ On-chip ratiometric calibration allows quantitative detection with minimal user intervention, and automated signal processing ensures reproducibility even in non-specialist settings. Field testing in various food matrices, including milk, seafood, and processed meats, has demonstrated promising recovery rates and low detection limits, confirming the feasibility of real-time surveillance. Sensor regeneration, storage stability, and robustness against photobleaching remain critical for repeated or prolonged use.

Successful translational implementation relies on a multidisciplinary approach, combining expertise in nanomaterials, analytical chemistry, regulatory compliance, and food technology. Collaborative validation studies, standardization of measurement protocols, and comprehensive training for operators facilitate the deployment of MQD-based sensors beyond the laboratory. When these translational barriers are addressed, MQD sensors provide rapid, cost-effective, and high-throughput solutions for food safety monitoring.^74,79^ They hold potential to enhance regulatory compliance, consumer protection, and supply chain transparency, bridging laboratory innovation and real-world applications with robust, deployable, and quantitative analytical platforms.

### 5.4. Stability, photobleaching, and reliability constraints in practical applications

Although MQDs have demonstrated excellent photophysical performance in controlled laboratory environments, their transition into field-deployable food safety sensors is often constrained by practical limitations associated with long-term stability, photobleaching resistance, and reproducibility under real-world conditions. The high surface reactivity of MQDs, while favorable for sensing, also makes them susceptible to oxidation, hydrolysis, or ligand desorption when exposed to variable pH, temperature, or oxygen levels commonly encountered in food matrices. These chemical transformations can lead to fluorescence drift, signal degradation, or even complete quenching over time, thereby restricting sensor shelf-life and operational reliability.^71,73^

Photobleaching remains another critical obstacle for MQD-based sensors, especially during prolonged optical excitation in fluorescence or electrochemiluminescence measurements. Continuous illumination can trigger surface oxidation or defect rearrangement, progressively diminishing luminescence intensity and altering ratiometric readouts. Protective strategies such as surface passivation, encapsulation within polymers or hydrogels, and the use of antioxidant additives can mitigate these effects; however, each introduces potential trade-offs between sensitivity, diffusion rate, and response time.

Furthermore, batch-to-batch variability continues to present a pressing challenge. Small fluctuations in precursor composition, etching environment, or termination density can yield substantial differences in emission wavelength and quantum yield, affecting analytical reproducibility across production lots.^75,77^ This variability complicates calibration, cross-laboratory comparisons, and quality assurance—factors that are central to regulatory validation and industrial deployment.

Addressing these stability- and reproducibility-related constraints requires integrating standardized synthesis protocols, rigorous photophysical benchmarking, and environmental stress testing into sensor development workflows. By quantifying degradation kinetics and implementing materials-level stabilization approaches, MQD-based food safety sensors can move closer to dependable, regulatory-compliant operation in complex, real-world monitoring scenarios.

## 6. Conclusion

MQDs have emerged as versatile nanomaterials for fluorescent and ratiometric sensing, offering remarkable sensitivity, tunable optical properties, and facile functionalization for the detection of diverse food contaminants. This review highlights the evolution of MQD-based sensors from fundamental photophysical investigations to advanced multi-analyte detection platforms capable of addressing biogenic amines, antibiotics, and metal ions in complex food matrices. Functionalization strategies, including heteroatom doping and molecular imprinting, significantly enhance selectivity, signal modulation, and compatibility with portable detection systems. Comparative analysis of reported analytical performance demonstrates that MQDs can achieve low detection limits, broad linear ranges, and satisfactory reproducibility, highlighting their strong potential for real-time and *in situ* food safety monitoring.

Despite these promising advances, several challenges must be addressed to facilitate the broader implementation of MQD-based sensing technologies. Translational considerations such as scalable and controllable synthesis, batch-to-batch reproducibility, and standardized characterization protocols remain critical for bridging the gap between laboratory-scale demonstrations and practical sensing platforms. In addition, further studies are required to clarify the fundamental photophysical mechanisms governing fluorescence emission, energy transfer, and quenching processes in MQDs, which are essential for rational sensor design and performance optimization.

Future research should also prioritize the development of robust sensing architectures capable of operating reliably in complex food matrices. Integration with portable and user-friendly platforms, including paper-based devices, microfluidic systems, and smartphone-assisted detection technologies, will be important for enabling on-site monitoring and rapid decision-making. Furthermore, improving selectivity toward structurally similar analytes and minimizing matrix interference remain key challenges for real-world applications.

Another important research direction involves comprehensive evaluation of toxicity, environmental impact, and long-term stability of MQDs, particularly in food-related contexts. Strategies such as surface engineering, immobilization on solid substrates, and environmentally benign synthesis routes may help improve the safety and sustainability of MQD-based sensing systems.

Overall, continued interdisciplinary efforts integrating materials science, analytical chemistry, nanotechnology, and food safety research will be essential to unlock the full potential of MQD-based fluorescent sensors. By addressing current challenges and focusing on the outlined research priorities, MQDs may evolve into practical and reliable tools for rapid, high-throughput, and field-deployable food contaminant detection, ultimately contributing to improved food quality control and public health protection.

## Conflicts of interest

The authors declare that they have no known competing financial interests or personal relationships that could have appeared to influence the work reported in this paper.

## Data Availability

Data sharing not applicable to this article as no datasets were generated or analysed during the current study.
